# White‐tailed deer exploit temporal refuge from multi‐predator and human risks on roads

**DOI:** 10.1002/ece3.9125

**Published:** 2022-07-24

**Authors:** Todd M. Kautz, Nicholas L. Fowler, Tyler R. Petroelje, Dean E. Beyer, Jared F. Duquette, Jerrold L. Belant

**Affiliations:** ^1^ College of Environmental Science and Forestry, Global Wildlife Conservation Center State University of New York Syracuse New York USA; ^2^ Wildlife Division Michigan Department of Natural Resources Lansing Michigan USA; ^3^ Department of Fisheries and Wildlife Michigan State University East Lansing Michigan USA

**Keywords:** *Canis latrans*, *Canis lupus*, functional diversity, *Lynx rufus*, *Odocoileus virginianus*, predation, temporal partitioning, *Ursus americanus*

## Abstract

Although most prey have multiple predator species, few studies have quantified how prey respond to the temporal niches of multiple predators which pose different levels of danger. For example, intraspecific variation in diel activity allows white‐tailed deer (*Odocoileus virginianus*) to reduce fawn activity overlap with coyotes *(Canis latrans*) but finding safe times of day may be more difficult for fawns in a multi‐predator context. We hypothesized that within a multi‐predator system, deer would allocate antipredation behavior optimally based on combined mortality risk from multiple sources, which would vary depending on fawn presence. We measured cause‐specific mortality of 777 adult (>1‐year‐old) and juvenile (1–4‐month‐old) deer and used 300 remote cameras to estimate the activity of deer, humans, and predators including American black bears (*Ursus americanus*), bobcats (*Lynx rufus*), coyotes, and wolves (*Canis lupus*). Predation and vehicle collisions accounted for 5.3 times greater mortality in juveniles (16% mortality from bears, coyotes, bobcats, wolves, and vehicles) compared with adults (3% mortality from coyotes, wolves, and vehicles). Deer nursery groups (i.e., ≥1 fawn present) were more diurnal than adult deer without fawns, causing fawns to have 24–38% less overlap with carnivores and 39% greater overlap with humans. Supporting our hypothesis, deer nursery groups appeared to optimize diel activity to minimize combined mortality risk. Temporal refuge for fawns was likely the result of carnivores avoiding humans, simplifying diel risk of five species into a trade‐off between diurnal humans and nocturnal carnivores. Functional redundancy among multiple predators with shared behaviors may partially explain why white‐tailed deer fawn predation rates are often similar among single‐ and multi‐predator systems.

## Graphical abstract

We assessed how adult and juvenile white‐tailed deer balanced temporal overlap with humans and 4 species of carnivores, when the level of threat posed depended on deer age and the species encountered. Juvenile deer in particular exploited a "safe" temporal window in the morning and evening hours, which avoided peak human activity but capitalized on suppression of carnivores by moderate human activity. This behavior appeared optimal from radio‐collared fawn mortality, where vehicle collisions killed some fawns but were far outweighed by the combined predation of 4 carnivore species.

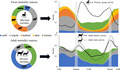

## 1 INTRODUCTION

Predation is the most common source of mortality among the world's wild terrestrial vertebrates, followed by human harvest and vehicle collisions (Hill et al., 2019). Prey respond to mortality risk from predators or humans using antipredator behaviors including vigilance, avoidance, and stealth (Brown et al., 1999; Frid & Dill, 2002). Risk responses can reduce predation and facilitate prey coexistence with diverse carnivore assemblages (Owen‐Smith, 2015) and have important ecosystem effects by changing herbivore foraging behavior (Creel & Christianson, 2008; Owen‐Smith, 2019). However, ungulate risk effect studies have usually focused on single predator‐single prey relationships despite multi‐predator systems being more common (Montgomery et al., 2019; Prugh et al., 2019). Consequently, there is a need to understand how ungulates balance antipredator behaviors when confronted by risks from multiple species.

In multi‐predator systems, animal responses to predation risk can vary with the extent of overlap among predator foraging strategies and relative risk of predators (Prugh et al., 2019). Functional redundancy (i.e., similar hunting patterns) among predator species can allow prey to use one risk response to avoid multiple predators, while functional divergence among predators can force prey to make trade‐offs in which avoiding one predator species requires increasing risk from another (Prugh et al., 2019). For example, some African ungulates adjust their predation risk by selecting for grasslands where coursing predators (wild dogs [*Lycaon pictus*] and cheetahs [*Acinonyx jubatus*]) tend to hunt, while avoiding brushy areas where ambush predators (lions [*Panthera leo*] and leopards [*Panthera pardus*]) tend to hunt (Thaker et al., 2011). In this example, functional redundancy facilitates avoidance of multiple ambush predators, but functional divergence causes brush and grasslands to each contain specialized predators. The spatial risk trade‐off confronting prey is, therefore, not determined by any single predator, but rather the combined risk from all predators within brush and grassland areas. A similar pattern applies to temporal hunting strategies, where prey may alter their temporal overlap among diurnal, nocturnal, and crepuscular predators (Creel et al., 2019). When confronted with functionally diverse predators, prey would be expected to temporally avoid predators representing greater risk. However, few studies have quantified mortality risk and antipredation behaviors of prey in diverse predator guilds, and of those, support for a relationship between direct mortality and avoidance remains equivocal (Creel et al., 2019; Dröge et al., 2017).

Ungulate mortality is typically greatest within 4 months of birth, due mostly to predation (Gingery et al., 2018; Linnell et al., 1995). The juvenile life stage is also typified by the greatest number of predator species, when small body mass and limited mobility of juveniles allow smaller predators (e.g., red fox [*Vulpes vulpes*]) or less agile predators (e.g., bears [*Ursus spp*.]) to kill ungulates rarely captured as adults (Gervasi et al., 2012; Linnell et al., 1995; Zager & Beecham, 2006). Many ungulate species employ a “hider” strategy to protect offspring where juveniles spend extended periods in hiding while their mother forages nearby (Byers, 1997; Costelloe & Rubenstein, 2015; Haskell et al., 2010; Lent, 1974; Ozoga et al., 1982). The ability of juveniles to hide while their mothers forage is an important defense because although predators can locate stationary juveniles (Boone, 2019), juveniles have greater predation risk when active (Byers, 1997, Costelloe & Rubenstein, 2015; *but see* Chitwood et al., 2017). Typically, pregnant females of species employing the hider strategy will separate from adult males before parturition, and after parturition, offspring will transition from spending most of their time hiding to most of their time following their mother (Lent, 1974). Consequently, ungulates that use a hider strategy can have independent diel activity between adult males and females and partially independent activity between adult females and juveniles.

White‐tailed deer (*Odocoileus virginianus*; hereafter deer) adults and juveniles experience differing predation risks (Chitwood et al., 2015) and respond differently to predation risk (Gulsby et al., 2018). Demographic variability in diel activity may be an important predator defense for deer, which reduce nocturnal activity of young fawns to reduce their exposure to coyotes (*Canis latrans*; Higdon et al., 2019, Crawford et al., 2021) and when exposed to wolf urine (Palmer et al., 2021). However, these studies examined deer temporal responses to a single predator, whereas fawns in most deer populations are killed by multiple predators and humans (Gingery et al., 2018). Identifying diel periods of low risk would be more challenging in multi‐predator systems because sympatric carnivores generally have divergent diel activity (Botts et al., 2020; Hayward & Slotow, 2009; Shores et al., 2019). Human activity may also impact deer diel risk through direct mortality where human and deer activity overlap (e.g., vehicle collisions) or indirectly if avoiding humans alters diel activity overlap between deer and predators (Gaynor et al., 2018; Patten et al., 2019). One study has examined diel activity of fawns and multiple predators, finding that human activity spatiotemporally compressed fawn and predator interactions as these species of wildlife were driven to converging places and times of refuge from humans (Murphy et al., 2021). This suggests that human activities may drive increased fawn predation, which is contrary to general trends of greater fawn mortality in more forested, less agricultural landscapes (Gingery et al., 2018). Consequently, more research is needed to describe spatiotemporal interactions of deer, human, and multi‐predator systems.

We examined the influence of multispecies risk on diel activity of white‐tailed deer in the Upper Peninsula of Michigan, USA, during July–September when fawns are 1–4 months old. White‐tailed deer fawns spend much of their time bedded in hiding until about 12 weeks old (Huegel, 1985), with mothers usually remaining near their fawns and making frequent visits to provide care and nurse fawns (Ozoga et al., 1982). However, non‐breeding adult females (e.g., 1‐year‐old deer; Ozoga, 1987) and those losing fawns soon after birth (Kautz et al., 2019) may behave similarly to adult male deer, which provide no parental care. We hypothesized that deer would allocate antipredation behavior optimally based on combined mortality risk from multiple sources, which would vary depending on fawn presence. To test our hypothesis, we measured demographic‐specific predation rates of deer along with diel activity of deer, four carnivores, and humans. We predicted: (1) deer would increase activity during diel periods with lowest combined mortality risk; (2) fawns would have greater predation rates and diel risk avoidance than adult deer and constrain adult female activity when present; and (3) as fawn predation declines from mid to late summer (Kautz et al., 2019), fawns may increase their temporal overlap with predators.

## 2 METHODS

### 2.1 Site description

**FIGURE 1:**
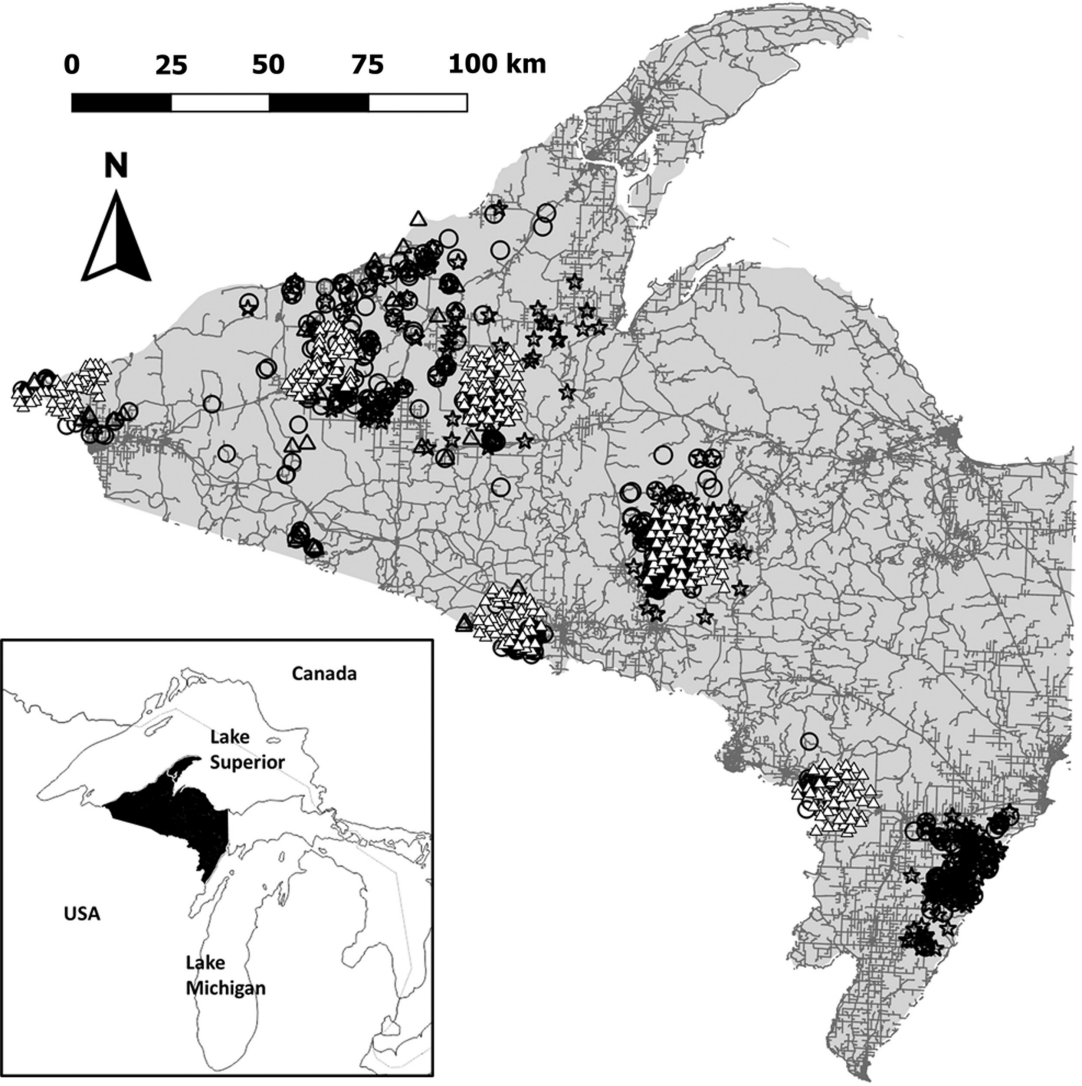
Locations of 777 radio‐collared white‐tailed deer (open triangles = adult males, open circles = adult females, open stars = fawns), and 300 remote cameras (white triangles), western Upper Peninsula of Michigan, USA (46.54° N, 88.77° W), July–September 2009–2019. Gray lines represent roads

We conducted our study in the western Upper Peninsula of Michigan (46.54 N, 88.77 W; Figure 1). The Upper Peninsula is bounded by Lake Superior on the north and Lake Michigan and Wisconsin on the south and is predominantly forested (85.2%; 2016 National Land Cover Database, Yang et al., 2018). Average road density is 0.69 km/km^2^ (U.S. Census Bureau, 2010). Average human population density is 8.76 humans/km^2^ and largely concentrated in towns with 61% of the area having ≤1 human resident/km^2^ (Center for International Earth Science Information Network, 2018). Important predators of juvenile deer in this area are wolves (*C. lupus*), coyotes, bobcats (*Lynx rufus*), and American black bears (*Ursus americanus*), with wolves and coyotes also potential predators of adult deer during summer, and vehicle collisions a risk to all deer (Duquette, 2014; Kautz et al., 2019). Deer hunting seasons were closed during our study period (July–September) but were open generally during October–December with most harvest consisting of adult males.

### 2.2 Measuring deer mortality

Deer in the Upper Peninsula migrate seasonally in local populations with fidelity to winter and summer ranges (Van Deelen et al., 1998). We captured deer throughout the western Upper Peninsula during 2009–2019, sampling adult females from 7 wintering populations, adult males from 5 wintering populations, and juvenile fawns from 3 summer populations (Figure 1). We captured adult deer during February–April using Clover traps (Clover, 1956) and Stevenson box traps (Anderson & Nielsen, 2002). Winter captures included 6–9‐month‐old deer, which were 1 year old before entering our adult survival sample in July. We manually restrained or chemically immobilized adult deer (Duquette et al., 2013) and fitted each with a VHF collar (Model M2510B; Advanced Telemetry Systems, Isanti, MN, USA) or satellite‐linked GPS collar (Plus Survey 1D collars, Vectronic Aerospace GmbH, Berlin, Germany). In the 3 populations where we collared neonatal fawns, we fit pregnant females with vaginal implant transmitters to locate parturition sites (model 3930, Advanced Telemetry Systems Inc.; Kautz et al., 2019). We captured fawns during May–July using systematic searches at birth sites and from opportunistic fawn encounters. We fitted fawns with expandable VHF radio‐collars (Model M2410; Advanced Telemetry Systems). We received mortality notifications via satellite from GPS collars and monitored VHF‐collared deer every 24–48 h during July–August and then twice weekly during September. We determined the cause of mortalities following Kautz et al. (2019). Animal handling procedures were approved by the Institutional Animal Care and Use Committees of Mississippi State University, Mississippi State, MS, USA (protocols 12–012, 15–013, 17–119), and State University of New York College of Environmental Science and Forestry (protocol 180,505).

We estimated cumulative incidence of deer mortality for radio‐collared fawns, adult (≥1‐year‐old) females, and adult (≥1‐year‐old) males during July 15–September 30 within competing risk agents including: anthropogenic (i.e., vehicle collision), coyote predation, bear predation, wolf predation, bobcat predation, and unidentified predation (predation events where the predator species could not be determined) using the Aalen–Johansen estimator in package “survival” (Therneau & Lumley, 2015) in program R (ver. 3.6.2, R Core Team, 2020), which accounts for right‐censoring for mortality from competing causes (Borgan, 1997). We used a daily survival step interval. Because no deer were captured during the survival interval, we did not use staggered entry. We right‐censored deer on the last known day alive if we were no longer able to detect the collar signal or recovered a collar that apparently slipped off the deer. Using fawns captured during opportunistic encounters can result in biased mortality estimates by missing mortality that happens shortly after birth (Gilbert et al., 2014), but this bias would not present during our monitoring period after July 15 as all fawns in the study were >10 days old by this time.

### 2.3 Measuring deer and predator activity

We evaluated deer and predator activity using remote cameras at 300 sites during July 15–September 15, 2017–2019. Camera surveys were designed to evaluate fawn recruitment at weaning, which is why we did not place cameras earlier in the summer to evaluate fawn risks and behaviors at <1 month old. Most cameras were operable on July 15 and all by August 1 each year. We placed cameras along unpaved roads including gravel roads, logging roads, or off‐highway vehicle trails. We deployed 48–52 cameras in each of 6 arrays placed on the summer ranges of 6 local populations where we had radio‐collared deer (Figure 1). Within each array, we created 2.25‐ × 2.25‐km cells and chose one camera location in each cell favoring accessible locations on public land as close to the cell centroid as practicable. For site independence, we used a minimum distance of 1.2 km among sites that exceeded the mean radius of late‐summer home ranges of adult female deer (Kautz et al., 2019). We attached one camera (Model sn84G or STC‐G45NG, Stealth Cam, Grand Prairie, TX, USA) to a tree 50–70 cm above ground and 3–5 m from road center, programmed to obtain 3 images for each detection with a 5‐s delay between detections.

From the camera survey, we considered all images of a single species within 15 minutes of separation at a site as a single detection. We classified deer demographic groups following Crawford et al. (2021) and Higdon et al. (2019), where any deer detection with at least one fawn present was considered a nursery group, an adult female detection with no fawns observed was classified as an adult female, and an adult male detection with no fawn present was classified as an adult male. We estimated diel activity distributions for deer nursery groups, adult female deer, adult male deer, coyotes, wolves, black bears, bobcats, and humans using a circular kernel density estimator within the Activity package (Rowcliffe, 2019) for program R. We assessed activity overlap (${\hat{\Delta }}_{4}$) of deer and predators using the compareCkern function (Ridout & Linkie, 2009; Rowcliffe, 2019).

### 2.4 Estimating diel response to predation risk

To evaluate deer activity in response to mortality risks from all predators and humans, we created a diel index of total risk as the sum of kernel density activity estimates for each predator species and humans weighted by their proportional contribution to the cumulative incidence of collared fawn mortality (e.g., a predator species that accounted for 40% of known‐cause mortality would have a weight of 0.4). We then replicated risk weights for adult deer based on mortality of radio‐collared adult deer. To test for changes in deer‐predator activity overlap through late summer, we calculated activity overlap between deer and carnivores (all four species combined) using a daily 11‐day moving window (date ±5 days) from July 20 to September 10.

## 3 RESULTS

**FIGURE 2:**
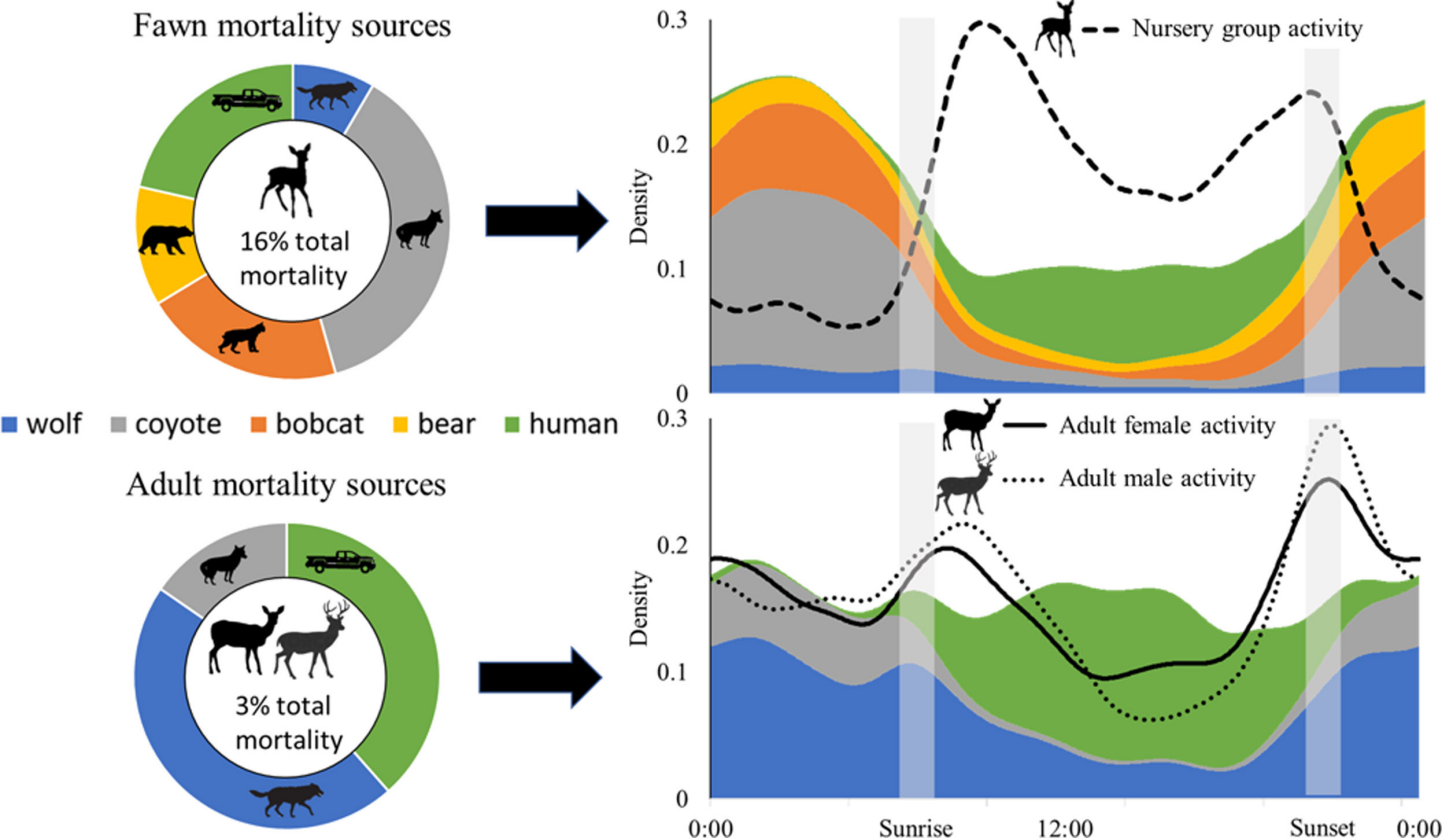
Temporal risk index for white‐tailed deer based on cause‐specific mortality, western Upper Peninsula of Michigan, USA, July–September 2009–2019. Pie charts on left represent proportional sources of predation and anthropogenic mortality for radio‐collared fawn and adult deer (males and females combined), excluding mortality from disease and unknown causes. Plots on right represent diel activity of fawn and adult deer (black lines), with a combined temporal risk index derived by multiplying carnivore and human activity by their proportional contributions to fawn and adult mortality (area plots with color correspond to predator species). Shaded gray areas reflect range of sunrise and sunset times during the study

We monitored the survival of 777 deer including 232 fawns (96 females, 136 males) and 545 adults (437 females and 108 males). Mean fawn birth date was June 7 (range = May 18–July 4); thus, our study period on average represented fawn survival from 5 to 16 weeks old. We observed 35 fawn and 15 adult deer mortalities. Additionally, 15 fawns were right‐censored before the end of monitoring because their collars fell off or we lost radio contact. Fawn mortality (16%) was 5.3 times greater than adult deer mortality (3%), which was similar between females (3%) and males (2%). For fawns and adult deer, no single cause accounted for >50% of mortality (Table 1, Figure 2). Fawns were killed most often by coyotes, followed by humans, bobcats, black bears, and wolves, while adult deer were killed by wolves, humans, and coyotes. Combined, humans and carnivores represented 94% of fawn and 87% of adult mortalities where a cause could be determined, with remaining deer mortality of known causes attributed to disease. We identified a probable predator species for all adult deer predations, but 20% of fawn mortalities were attributed to unidentified predators. In most cases, unidentified predation events could not be attributed to one species (e.g., black bear and coyote sign present), though by a species included in our study; we found no evidence that any other species killed deer. All anthropogenic deer mortality was from vehicle collisions.

**TABLE 1:** Predation rates and predator activity overlap of fawns and adult white‐tailed deer, Western Upper Peninsula of Michigan, USA, 2009–2019

Species	Fawn mortality rate	Adult mortality rate	Fawn risk weight	Adult risk weight	${\hat{\Delta }}_{4}$ nursery groups	${\hat{\Delta }}_{4}$ adult deer
American black bear	0.01	0.00	0.12	0.00	0.65 (0.64–0.65)	0.85 (0.85–0.86)
Bobcat	0.02	0.00	0.21	0.00	0.44 (0.43–0.46)	0.70 (0.69–0.71)
Coyote	0.04	<0.01	0.37	0.15	0.41 (0.40–0.42)	0.66 (0.65–0.66)
Wolf	0.01	0.01	0.08	0.46	0.61 (0.60–0.62)	0.86 (0.85–0.87)
Human	0.03	0.01	0.21	0.38	0.71 (0.71–0.72)	0.51 (0.51–0.51)

*Note*: Mortality rates reflect Aalen–Johannsen estimates for cumulative incidence of mortality by predator species for radio‐collared white‐tailed deer fawns (*n* = 230) and adult deer (*n* = 545) during 2009–2019. Risk weights were calculated as the proportion of identified predator and human‐caused mortality attributed to each predator species. Temporal overlap (${\hat{\Delta }}_{4}$) reflects the circular kernel density overlap between diel activity of deer and predators with 95% confidence limits, derived from remote camera observations during 2017–2019.

**FIGURE 3:**
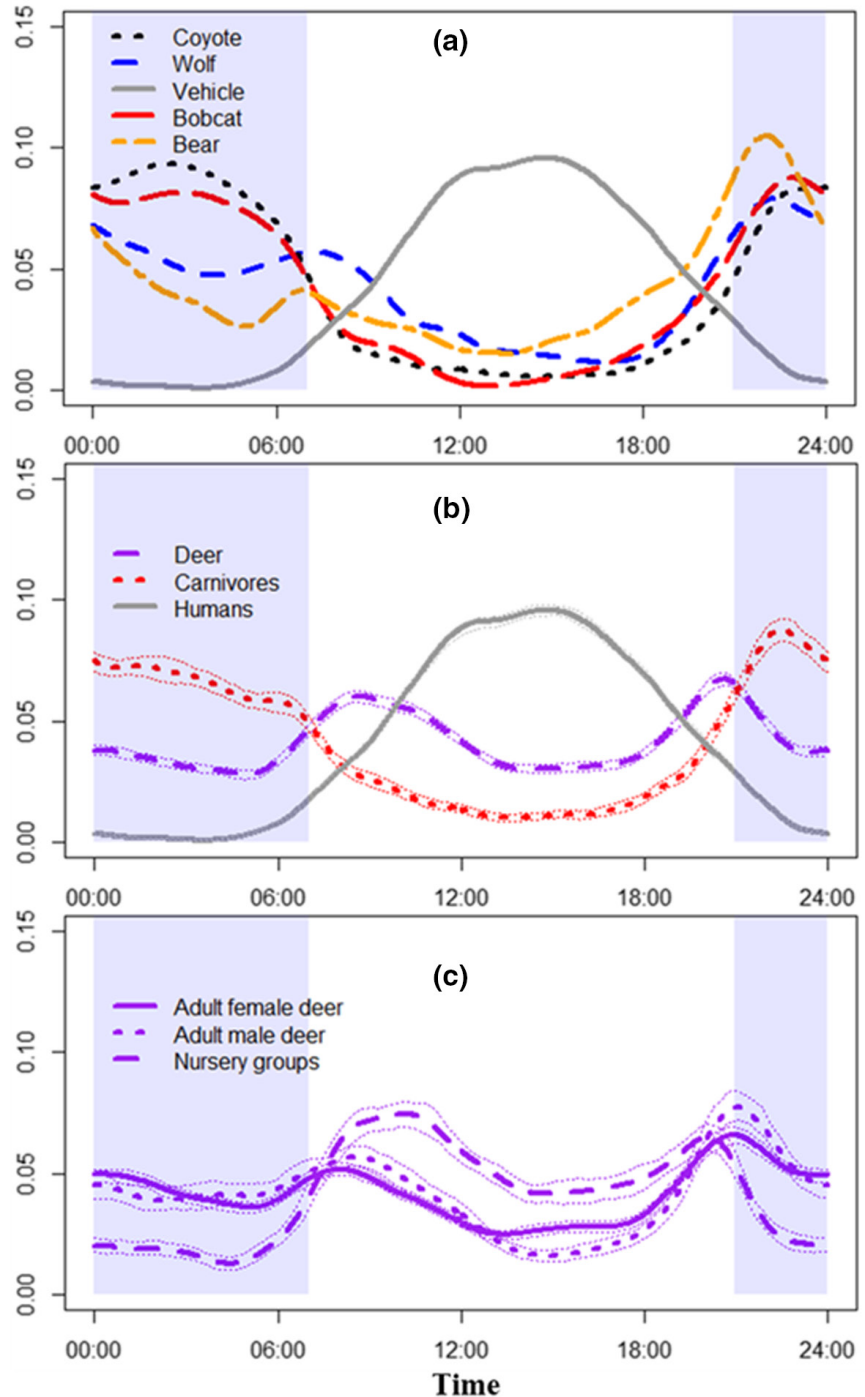
Diel activity estimates for (a) humans and carnivores; (b) humans, white‐tailed deer (all demographic groups combined), and combined carnivores (American black bear, bobcat, coyote, and wolf); and (c) white‐tailed deer by demographic group (adult female and male deer do not include detections with fawns present), western Upper Peninsula of Michigan, USA, July–September 2017–2019. Fine dotted lines represent 95% confidence limits. Shaded areas represent average nocturnal period (sunset to sunrise)

We recorded 16,193 independent detections of deer including 7379 adult females, 2438 adult males, and 3596 nursery groups over 34,810 monitoring days with remote cameras. Fawns were present in 26% of adult female detections and 6% of adult male deer detections. We also detected 1387 black bears, 356 bobcats, 2781 coyotes, 1400 wolves, and 27,228 humans. Overall, all four carnivores were nocturnal, humans were diurnal, and deer had crepuscular activity peaks (Figure 3). Adult male and adult female deer had similar temporal activity (${\hat{\Delta }}_{4}$ = 0.93), with adult males slightly more nocturnal. However, diel activity differed between nursery groups and adult deer (${\hat{\Delta }}_{4}$ = 0.74) by being primarily diurnal (Figure 2). When human and carnivore activity was weighted by contribution to deer mortality, day appeared the least risky period for fawns to use roads because of low activity from black bears, bobcats, coyotes, and wolves.

As a result of greater diurnal activity, nursery group overlap with carnivores was less than that of adult deer by 24%, 37%, 38%, and 29% for black bears, coyotes, bobcats, and wolves, respectively. However, nursery groups overlapped with humans 39% more than did adult deer (Table 1). Average overlap with humans and carnivore species combined was 56% for nursery groups and 72% for adult deer. Nursery group overlap with humans and carnivores decreased slightly during late July and remained stable through mid‐September (Figure S1).

## 4 DISCUSSION

We hypothesized that white‐tailed deer allocate antipredation behavior optimally based on combined mortality risk from multiple sources, which varies with fawn presence. Supporting our hypothesis, fawns had greater predation and anthropogenic mortality risk than adult deer and nursery group activity was concentrated during times of day when the most prominent fawn mortality sources were least active. Specifically, diel risk from five species responsible for fawn mortality was functionally simplified into nocturnal carnivores and diurnal humans, and deer nursery groups shifted toward a diurnal threat (vehicle collisions) of lower risk than the combined risk from four nocturnal carnivores. For adult deer without fawns, mortality risk was low and may not have triggered an avoidance response within diel activity.

Many ungulate populations experience mortality from multiple carnivores in addition to humans (Montgomery et al., 2019), and predators are especially diverse for juveniles (Gingery et al., 2018; Griffin et al., 2011; Linnell et al., 1995). Consequently, high‐ and low‐risk demographics may occur within the same ungulate population and respond differently to predators. Individuals with low risk of predation (i.e., lone adults or adult‐only groups) may prioritize foraging by being active throughout the diel period while high‐risk individuals (i.e., juveniles and juvenile‐containing groups) temporally avoid predators. Our results suggest that while deer can structure fawn temporal activity to reduce risk where a single predator dominates mortality (Crawford et al., 2021; Higdon et al., 2019), deer also can temporally avoid combined fawn risk from multiple predators and humans.

Large carnivores and mesocarnivores often become more nocturnal in response to human disturbance (Gaynor et al., 2018; Smith et al., 2018). Though diel activity is only one of many aspects of carnivore niche, our results indicate the functional diversity of diel activity among carnivores using roads was remarkably low and predictable. Concurrent with our study, GPS‐collared carnivores in our study area were primarily nocturnal near roads, but in areas farther from roads bobcats, coyotes, and wolves had roughly equal activity during day and night while bears were mostly diurnal (Kautz et al., 2021). We did not have a measure of how proximity to road affected fawn activity, but if fawns temporally avoided human activity near roads, it was evidently to a much lesser degree than carnivores did. Our results, therefore, represent a unique case of deer‐predator‐human interactions that occur near roads; however, influence of roads on carnivore behavior can reduce ungulate predation at larger spatial extents (Berger, 2007). Additionally, it is notable that our camera sites were not on high‐traffic roads; cameras in our study averaged <1 human detection per day, suggesting low human activity could facilitate temporal refuge for prey. This hypothesis was supported in our study area by reduced fawn predation risk in areas with greater human development (Kautz, 2021). However, an interesting contrast to our results occurred in white‐tailed deer fawns in Pennsylvania, USA, where fawn‐predator temporal overlap was greater in a more developed landscape (Murphy et al., 2021).

We identified divergent diel activity patterns among deer age and sex classes, as found previously (Crawford et al., 2021; Higdon et al., 2019; Lashley et al., 2014; Murphy et al., 2021). Deer fawns in the southeastern United States increased diurnal activity compared with adult deer to avoid their primary predator, coyotes (Crawford et al., 2021, Higdon et al., 2019). Deer exposed to wolf urine in a wolf‐free environment become more diurnal (Palmer et al., 2021), suggesting that reduced nocturnal behavior is a general deer reaction to predation risk and not a response to nocturnal activity from sympatric predators. It would, therefore, be interesting to determine whether deer have the behavioral plasticity to shift fawn activity toward nocturnality if confronted by a primarily diurnal risk such as humans in a system lacking fawn predators.

Temporal partitioning during summer between males and females occurs in other deer populations, with females more diurnal (Biggerstaff et al., 2017). Our study corroborates these results but with an important caveat: adult female deer were more diurnal than males overall because females were more often accompanied by fawns (i.e., members of nursery groups), but adult male and female deer had similar activity patterns when fawns were absent. Therefore, temporal partitioning between adult male and female deer appeared mediated by limitations on maternal female activity imposed by fawn predation risk. Risk of offspring predation and nutritional demands of lactation cause spatial and dietary partitioning between adult male and female ungulates (Han et al., 2021; Loe et al., 2006; Main, 2008; Ruckstuhl & Neuhaus, 2002), so our results add to the broad effects of offspring care on intraspecific niche variation in ungulates.

Trade‐offs between predator avoidance and foraging may explain why predator activity had little apparent influence on adult deer activity. Most ungulates consume large amounts of widely dispersed food, making movement essential to foraging (Senft et al., 1987). Ungulates respond to long‐term predation risk by reducing movements (Dröge et al., 2019), but temporally restricting movement can negatively impact nutrition (Owen‐Smith, 2019), a central determinant of fitness for adult ungulates (Parker et al., 2009). A summer nutritional surplus is essential for adult deer in northern populations to improve winter survival and future reproduction (DelGiudice et al., 1992; Kautz et al., 2020; Mautz, 1978; Mech et al., 1991). Hence, adult deer unaccompanied by fawns in our study had potentially large fitness benefits from maximizing feeding time with low risk of predation during summer. For mothers accompanied by fawns, response to predators may shift as female ungulates also increase fitness by protecting their offspring (Lent, 1974; Main, 2008).

Prey likely respond differently to predator species based on relative risk but determining how prey prioritize response to predators is difficult. We used the number of deer killed by each species to index risk among predators, which is directly related to deer survival. However, a possible limitation of our approach is that realized predation rates may not represent inherent predation risk (i.e., the level of risk a predator would represent in the absence of prey antipredation behaviors) because if prey use greater effort to avoid a higher‐risk predator, that predator may kill few prey (Creel et al., 2019). The most effective antipredator behavior should prioritize predators based on inherent risk, which may be nearly impossible to measure in wild populations (Creel et al., 2019). A second potential limitation in using mortality rates to index risk is that prey behavior may be more influenced by perceived than actual risk (Gaynor et al., 2019). Consequently, the predator that kills the most prey may not always elicit the strongest antipredator response from ungulates (Creel et al., 2017). Despite potential limitations, our results supported risk avoidance in proportion to mortality rate as nursery groups avoided overlap with carnivores more than humans when carnivores killed four times more fawns than did humans.

Increasing predator diversity may not lead to a reduction in safe conditions for prey if predators share similar hunting styles or if interference among predators reduces hunting efficacy (Schmitz, 2007). Our results suggest the functional diversity of predators within a niche dimension may be surprisingly low if predators are driven to similar behaviors by a shared priority such as avoiding humans. Under such conditions, prey may be able to tailor responses to favor either human or carnivore encounters based on the needs and risks of each individual. Prey with especially high predation risk, such as neonatal white‐tailed deer, may accept more interaction with humans in exchange for reduced predator encounters. Human‐mediated redundancy among carnivores may allow deer to avoid a suite of predation risks with a single behavioral adaptation, which may in part explain why deer fawn predation rates are often similar across systems with 1–4 predator species (Gingery et al., 2018; Kautz et al., 2019; Shuman et al., 2017).

## AUTHOR CONTRIBUTIONS

**Todd M Kautz:** Conceptualization (equal); data curation (equal); formal analysis (lead); investigation (lead); methodology (lead); project administration (equal); writing – original draft (lead); writing – review and editing (lead). **Nicholas L Fowler:** Data curation (equal); formal analysis (supporting); investigation (equal); methodology (equal); project administration (equal); writing – original draft (supporting); writing – review and editing (supporting). **Dean E. Beyer, Jr.:** Conceptualization (equal); investigation (equal); methodology (equal); project administration (lead); resources (equal); supervision (equal); writing – original draft (supporting); writing – review and editing (supporting). **Jared F. Duquette:** Data curation (equal); investigation (equal); methodology (equal); project administration (equal); writing – original draft (supporting); writing – review and editing (supporting). **Tyler Petroelje:** Data curation (equal); investigation (equal); methodology (equal); project administration (equal); writing – original draft (supporting); writing – review and editing (supporting). **Jerrold L. Belant:** Conceptualization (equal); funding acquisition (lead); methodology (supporting); project administration (supporting); resources (lead); supervision (lead); writing – original draft (equal); writing – review and editing (equal).

## CONFLICT OF INTEREST

The authors have no competing interests to declare.

## Supporting information


Figure S1

## Data Availability

Data used in this manuscript will be made publicly available from the Dryad repository (https://doi.org/10.5061/dryad.70rxwdc0h) upon acceptance for publication.
