# Supplementary material for: White‐tailed deer exploit temporal refuge from multi‐predator and human risks on roads
Source: Ecol Evol. 2022 Jul 24;12(7):e9125. doi: 10.1002/ece3.9125 (PMC9309034; doi:10.1002/ece3.9125)
Supplement: Supplementary file 1 — Figure S1 [file ECE3-12-e9125-s001.docx]

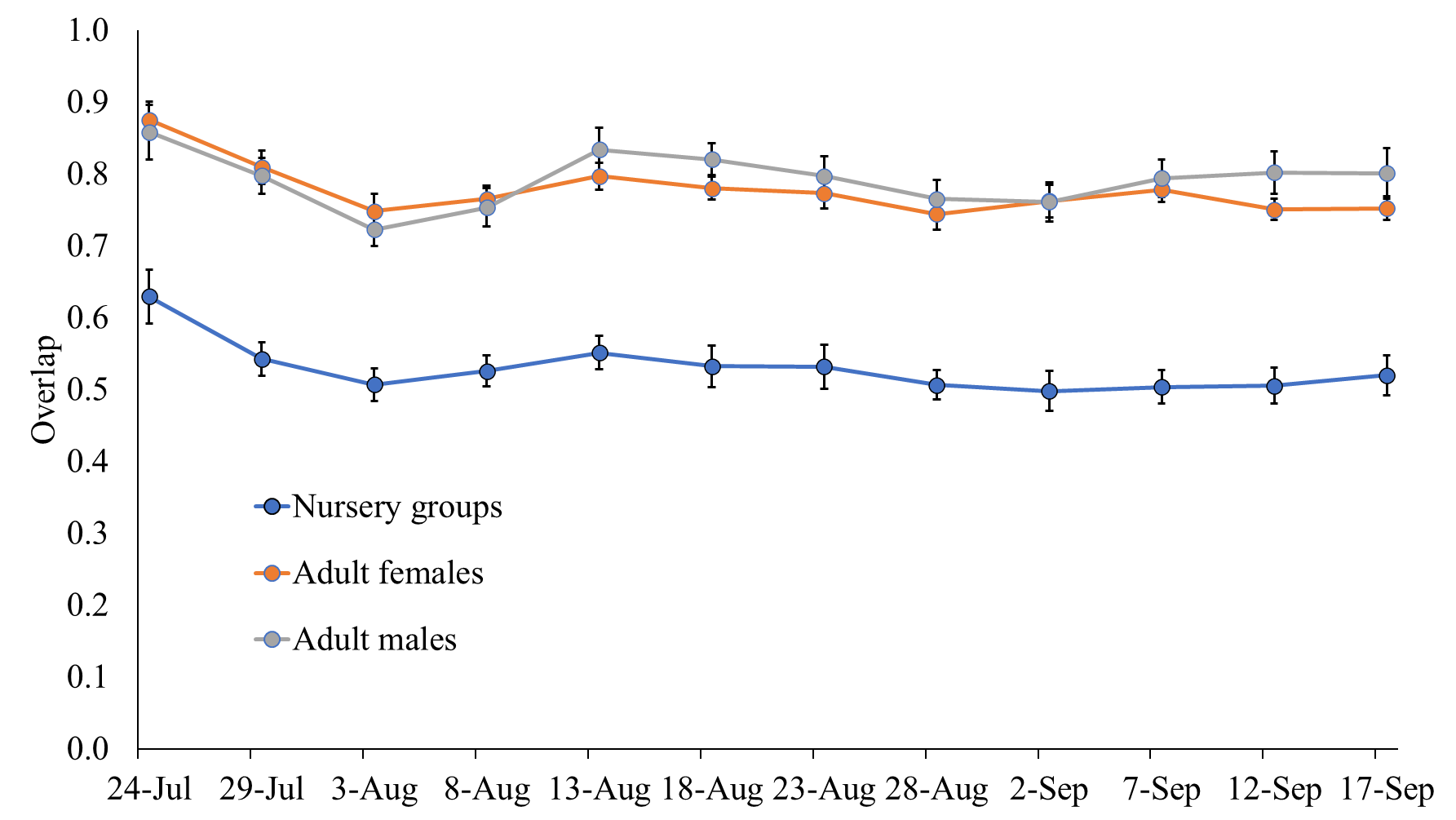


**Figure S1.** Diel activity overlap (
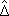
_4_) between deer and carnivores (American black bear, bobcat, coyote, and wolf) within 11-day intervals, Western Upper Peninsula of Michigan, USA, July-September 2017–2019. Error bars represent 95% confidence limits.
